# Supplementary material for: Prevalence of hepatitis B/C viruses and associated factors in key groups attending a health services institution in Colombia, 2019
Source: PLoS One. 2020 Sep 22;15(9):e0238655. doi: 10.1371/journal.pone.0238655 (PMC7508402; doi:10.1371/journal.pone.0238655)
Supplement: S1 File — (DOCX) [file pone.0238655.s001.docx]

**Supplementary material 1.**

**Prevalence of viral hepatitis type B and C - Survey**

We invite you to participate in the study entitled "Prevalence of viral hepatitis type B and C" led by the Fundación Antioqueña de Infectología. This work seeks to estimate the prevalence of both infections and identify the main risk factors in various population groups.

We ask you to indicate an option in each of the statements that appear in this survey, which will take between 2 and 5 minutes. The survey will be applied for a physician, but if you wish, you can also fill it out yourself (self-filled).

**Population group**

General ___ Men who have sex with other men (MSM) ___ Sex worker ___

Homeless person ___ Vulnerable youth ___ Inmate ___

Bisexual ___ Injecting drug user ___ Lesbian ___

Transsexual ___

**Origin (City** **where you currently reside)**

Medellín ___ Cali ___ Bogotá ___ Quibdó ___

Pereira ___ Other ___

**Sex**

Woman ___ Man ___

**Age group**

Adolescents (< 21 years) ___ 21–30 years ___ 31–40 years ___

41–50 years ___ 51–60 years ___ Older than 60 years ___

**Schooling**

None ___ Incomplete primary ___ Complete primary ___ Incomplete secondary ___ Completed secondary ___ Technical ___ University ___

**Marital status**

Married ___ Common Law Marriage ___ Single ___ Widowed ___ Separated ____

**Ethnicity (self-perceived)**

Afro-descendent ____ White ____ Mestizo ___

**Health affiliation**

Without affiliation ____ Subsidised regime ___ Contributory regime ___

**Health features**

|  | **Yes** | **Not** |
| --- | --- | --- |
| Hospitalisation in the last 12 months | Yes | Not |
| Medication in the last month | Yes | Not |
| Having received a transfusion or transplant | Yes | Not |
| Having been vaccinated within the last year | Yes | Not |
| Use of hallucinogens | Yes | Not |
| Risky sexual behaviour | Yes | Not |
| Intercourse with key group | Yes | Not |
| Intercourse with people with STIs | Yes | Not |
| New sexual partner within the last six (6) months | Yes | Not |
| Having received psychoactive substances or money in exchange for a intercourse | Yes | Not |
| In the last 12 months the subject or his sexual partner has been deprived of liberty | Yes | Not |
| In the last 12 months the subject or his sexual partner had piercings made | Yes | Not |
| In the last 12 months the subject has suffered biohazard accidents | Yes | Not |

**Prevalencia de las hepatitis virales tipo B y C - Encuesta**

Lo invitamos a participar del estudio titulado "*Prevalencia de las hepatitis virales tipo B y C*" liderado por la Fundación Antioqueña de Infectología. Este trabajo busca estimar la prevalencia de ambas infecciones e identificar los principales factores de riesgo en varios grupos poblaciones.

A continuación, le pedimos señalar sólo una opción en cada uno de los enunciados que aparecen en esta encuesta, cuyo diligenciamiento demora un tiempo entre 2 y 5 minutos. La encuesta será aplicada por un médico, pero si usted lo desea también puede diligenciarla usted mismo (autodiligenciada).

**Grupo poblacional**

General ___ Hombre que tiene sexo con otros hombres (MSM) ___ Trabajador(a) sexual ___

Habitante de calle ___ Joven vulnerable ___ Preso ___

Bisexual ___ Usuario de drogas inyectables ___ Lesbiana ___

Transexual ___

**Ciudad de Origen (donde reside actualmente)**

Medellín ___ Cali ___ Bogotá ___ Quibdó ___

Pereira ___ Otra ___

**Sexo**

Mujer ___ Hombre ___

**Grupo etario**

Adolescente (< 21 años) ___ 21–30 años ___ 31–40 años ___

41–50 años ___ 51–60 años ___ Mayor de 60 años ___

**Escolaridad**

Ninguna ___ Primaria Incompleta ___ Primaria Completa ___ Secundaria Incompleta ___ Secundaria Completa ___ Técnico ___ Universitario ___

**Estado civil**

Casado ___ Unión libre ___ Soltero ___ Viudo ___ Separado ____

**Etnia (auto-percibida)**

Afro-descendiente ____ Blanco ____ Mestizo ___

**Afiliación en salud**

Sin afiliación ____ Régimen Subsidiado ___ Régimen Contributivo ___

**Factores de salud**

|  | **Si** | **No** |
| --- | --- | --- |
| Ha sido hospitalizado los últimos 12 meses | Si | No |
| Recibió y consumió medicamentos el último mes | Si | No |
| Ha recibido una transfusión sanguínea o un trasplante | Si | No |
| Ha sido vacunado el último año | Si | No |
| Usa drogas alucinógenas (de manera frecuente el último año) | Si | No |
| Ha tenido intercambios sexuales de riesgo (el último año) | Si | No |
| Ha tenido intercambios sexuales con grupos clave (el último año) | Si | No |
| Ha tenido intercambios sexuales con personas con ITS (el último año) | Si | No |
| Ha tenido nuevo compañero sexual los últimos seis meses | Si | No |
| Ha recibido psicoactivos o dinero por tener un intercambio sexual (el último año) | Si | No |
| En los últimos 12 meses usted o su compañero sexual ha estado preso | Si | No |
| En los últimos 12 meses usted o su compañero sexual ha realizado perforaciones como piercing | Si | No |
| En los últimos 12 meses usted o su compañero sexual ha a tenido un accidente que implique riesgo biológico | Si | No |

Nota: Todas las variables se clasifican de naturaleza cualitativa y su definición operativa es la misma que aparece en cada enunciado.
