# Supplementary material for: Prevalence of hepatitis B/C viruses and associated factors in key groups attending a health services institution in Colombia, 2019
Source: PLoS One. 2020 Sep 22;15(9):e0238655. doi: 10.1371/journal.pone.0238655 (PMC7508402; doi:10.1371/journal.pone.0238655)
Supplement: Attachment — Submitted filename: PONE-D-20-11570_reviewer.pdf [file pone.0238655.s002.pdf]

PLOS ONE

Prevalence of hepatitis B/C viruses and associated factors in key groups attending a health services institution in Colombia, 2019

--Manuscript Draft--

Manuscript Number:	PONE-D-20-11570
Article Type:	Research Article
Full Title:	Prevalence of hepatitis B/C viruses and associated factors in key groups attending a health services institution in Colombia, 2019
Short Title:	Hepatitis B/C virus prevalence and factors in Colombia
Corresponding Author:	Jaiberth Antonio Cardona-Arias, MSc<br>Universidad de Antioquia<br>Medellín, Antioquia COLOMBIA
Keywords:	hepatitis B virus; Hepatitis C virus; prevalence; Risk factors; Colombia.
Abstract:	Both hepatitis B virus (HBV) and hepatitis C virus (HCV) are major sources of morbidity and mortality worldwide; however, their prevalence in key groups in Colombia is not yet known. We aimed to analyse the prevalence of HBV and HCV and its associated factors in key groups who were treated at an institution providing health services in Colombia during 2019. This was a multiple-group ecological study that included 2,624 subjects from the general population, 1,100 men who have had sex with men (MSM), 1,061 homeless individuals, 380 sex workers, 260 vulnerable young people, 202 drug users, 41 inmates and 103 people from the lesbian, gay, bisexual and transgender community. Prevalence of infection with a 95% confidence interval and its associated factors was calculated for each group. Confounding variables were assessed using logistical regression and SPSS 25.0 software. Prevalence of HBV and HCV in the general population was 0.15% and 0.27%, respectively; 0.27% and 2.09% in MSM; 0.37% and 2.17% amongst homeless individuals; 0.26% and 0.0% amongst sex workers; 0.39% and 0.0% amongst vulnerable youth; and 5.94% and 45.54 amongst injecting drug users. In the multivariate HBV model, the explanatory variables included the study group, city of origin and the type of health affiliation; for HCV they were group, origin, sex, age group, health affiliation, use of drugs and hallucinogen use during sexual intercourse. A high prevalence of HBV and HCV were evidenced for both viral infections, which was, consequently, much higher within the key groups. The main associated factors that were identified related to origin and type of health affiliation and demonstrated a double vulnerability, that is, belonging to groups that are discriminated and excluded from many health policies and living under unfavourable socioeconomic conditions that prevent proper affiliation and health care.
Order of Authors:	Jaiberth Antonio Cardona-Arias, MSc
	Juan Carlos Cataño Correa
	Luis Felipe Higuita-Gutiérrez
Additional Information:	
Question	Response
**Financial Disclosure**<br><br>Enter a financial disclosure statement that describes the sources of funding for the work included in this submission. Review the submission guidelines for detailed requirements. View published research articles from *PLOS ONE* for specific examples.<br><br>This statement is required for submission	The author(s) received no specific funding for this work.

and **will appear in the published article** if the submission is accepted. Please make sure it is accurate.<br><br>**Unfunded studies**<br>Enter: *The author(s) received no specific funding for this work.*<br><br>**Funded studies**<br>Enter a statement with the following details:<br>• Initials of the authors who received each award<br>• Grant numbers awarded to each author<br>• The full name of each funder<br>• URL of each funder website<br>• Did the sponsors or funders play any role in the study design, data collection and analysis, decision to publish, or preparation of the manuscript?<br>• **NO** - Include this sentence at the end of your statement: *The funders had no role in study design, data collection and analysis, decision to publish, or preparation of the manuscript.*<br>• **YES** - Specify the role(s) played.<br><br>* typeset	
**Competing Interests**<br><br>Use the instructions below to enter a competing interest statement for this submission. On behalf of all authors, disclose any competing interests that could be perceived to bias this work—acknowledging all financial support and any other relevant financial or non-financial competing interests.<br><br>This statement **will appear in the published article** if the submission is accepted. Please make sure it is accurate. View published research articles from *PLOS ONE* for specific examples.	The authors have declared that no competing interests exist.

**NO authors have competing interests**<br><br>Enter: *The authors have declared that no competing interests exist.*<br><br>**Authors with competing interests**<br><br>Enter competing interest details beginning with this statement:<br><br>*I have read the journal's policy and the authors of this manuscript have the following competing interests: [insert competing interests here]*<br><br>* typeset	
**Ethics Statement**<br><br>Enter an ethics statement for this submission. This statement is required if the study involved:<br><br>• Human participants<br>• Human specimens or tissue<br>• Vertebrate animals or cephalopods<br>• Vertebrate embryos or tissues<br>• Field research<br><br>Write "N/A" if the submission does not require an ethics statement.<br><br>General guidance is provided below. Consult the submission guidelines for detailed instructions. **Make sure that all information entered here is included in the Methods section of the manuscript.**	FAI reviewed the research and gave the ethical endorsement.<br>We include the statement indicating approval of this research<br>We indicate the form of consent obtained (written)

Powered by Editorial Manager® and ProduXion Manager® from Aries Systems Corporation

**Format for specific study types**<br><br>**Human Subject Research (involving human participants and/or tissue)**<br>• Give the name of the institutional review board or ethics committee that approved the study<br>• Include the approval number and/or a statement indicating approval of this research<br>• Indicate the form of consent obtained (written/oral) or the reason that consent was not obtained (e.g. the data were analyzed anonymously)<br><br>**Animal Research (involving vertebrate animals, embryos or tissues)**<br>• Provide the name of the Institutional Animal Care and Use Committee (IACUC) or other relevant ethics board that reviewed the study protocol, and indicate whether they approved this research or granted a formal waiver of ethical approval<br>• Include an approval number if one was obtained<br>• If the study involved *non-human primates*, add *additional details* about animal welfare and steps taken to ameliorate suffering<br>• If anesthesia, euthanasia, or any kind of animal sacrifice is part of the study, include briefly which substances and/or methods were applied<br><br>**Field Research**<br><br>Include the following details if this study involves the collection of plant, animal, or other materials from a natural setting:<br>• Field permit number<br>• Name of the institution or relevant body that granted permission	
**Data Availability**<br><br>Authors are required to make all data underlying the findings described fully available, without restriction, and from the time of publication. PLOS allows rare exceptions to address legal and ethical concerns. See the PLOS Data Policy and FAQ for detailed information.	Yes - all data are fully available without restriction

Powered by Editorial Manager® and ProduXion Manager® from Aries Systems Corporation

A Data Availability Statement describing where the data can be found is required at submission. Your answers to this question constitute the Data Availability Statement and **will be published in the article**, if accepted.<br><br>**Important:** Stating 'data available on request from the author' is not sufficient. If your data are only available upon request, select 'No' for the first question and explain your exceptional situation in the text box.<br><br>Do the authors confirm that all data underlying the findings described in their manuscript are fully available without restriction?	
**Describe where the data may be found in full sentences. If you are copying our sample text, replace any instances of XXX with the appropriate details.**<br><br>• If the data are **held or will be held in a public repository**, include URLs, accession numbers or DOIs. If this information will only be available after acceptance, indicate this by ticking the box below. For example: *All XXX files are available from the XXX database (accession number(s) XXX, XXX.).*<br>• If the data are all contained **within the manuscript and/or Supporting Information files**, enter the following: *All relevant data are within the manuscript and its Supporting Information files.*<br>• If neither of these applies but you are able to provide **details of access elsewhere**, with or without limitations, please do so. For example:<br><br>*Data cannot be shared publicly because of [XXX]. Data are available from the XXX Institutional Data Access / Ethics Committee (contact via XXX) for researchers who meet the criteria for access to confidential data.*<br><br>*The data underlying the results presented in the study are available from (include the name of the third party*	All relevant data are within the manuscript and its Supporting Information files.

and contact information or URL).<br>• This text is appropriate if the data are owned by a third party and authors do not have permission to share the data.<br><br>* typeset	
Additional data availability information:	

Powered by Editorial Manager® and ProduXion Manager® from Aries Systems Corporation

Manuscript

Click here to access/download;Manuscript;Manuscript II_
Prevalence HBV HCV Colombia.docx

Prevalence of hepatitis B/C viruses and associated factors in key groups attending a health services institution in Colombia, 2019

Short Title: Hepatitis B/C virus prevalence and factors in Colombia

Jaiberth Antonio Cardona-Arias[1]*, Juan Carlos Cataño Correa[2], Luis Felipe Higuita-Gutiérrez[3]

[1] Universidad de Antioquia, Medellín, Colombia

[2] Fundación Antioqueña de Infectología, Medellín, Colombia

[3] Universidad Cooperativa de Colombia, Medellín, Colombia

* Corresponding author:

E-mail: jaiberth.cardona@udea.edu.co (JACA)

Abstract

Both hepatitis B virus (HBV) and hepatitis C virus (HCV) are major sources of morbidity and mortality worldwide; however, their prevalence in key groups in Colombia is not yet known. We aimed to analyse the prevalence of HBV and HCV and its associated factors in key groups who were treated at an institution providing health services in Colombia during 2019. This was a multiple-group ecological study that included 2,624 subjects from the general population, 1,100 men who have had sex with men (MSM), 1,061 homeless individuals, 380 sex workers, 260 vulnerable young people, 202 drug users, 41 inmates and 103 people from the lesbian, gay, bisexual and transgender community. Prevalence of infection with a 95% confidence interval and its associated factors was calculated for each group. Confounding variables were assessed using logistical regression and SPSS 25.0 software. Prevalence of HBV and HCV in the general population was 0.15% and 0.27%, respectively; 0.27% and 2.09% in MSM; 0.37% and 2.17% amongst homeless individuals; 0.26% and 0.0% amongst sex workers; 0.39% and 0.0% amongst vulnerable youth; and 5.94% and 45.54 amongst injecting drug users. In the multivariate HBV model, the explanatory variables included the study group, city of origin and the type of health affiliation; for HCV they were group, origin, sex, age group, health affiliation, use of drugs and hallucinogen use during sexual intercourse. A high prevalence of HBV and HCV were evidenced for both viral infections, which was, consequently, much higher within the key groups. The main associated factors that were identified related to origin and type of health affiliation and demonstrated a double vulnerability, that is, belonging to groups that are discriminated and excluded from many health policies and living under unfavourable socioeconomic conditions that prevent proper affiliation and health care.

Introduction

Viral hepatitis, especially types B and C, which account for more than 95% of deaths in the population, generate a high morbidity and mortality burden worldwide, with figures ~~similar~~ to those from tuberculosis or HIV/AIDS, but with less investment in diagnosis, prevention and treatment. The most concerning data are those related with hepatitis C virus (HCV) and hepatitis B virus (HBV). In the case of HCV, due to an increase in its morbidity with a worldwide prevalence of 1% [1,2], there are currently 75-85% of cases corresponding to chronic infection, 10-20% at risk for developing cirrhosis within 30 years and a 1- 5% risk of hepatocellular carcinoma [3,4]. Furthermore, there are estimates of 1.75 million cases of HCV per year, however, due to low levels of screening most of the individuals infected are unaware of their serological status. This constitutes a serious clinical and public health issue due to an increased risk of transmission and progression to cirrhosis, hepatocellular carcinoma and death [1].

Similar? the mortality burden assoc to hepatitis viruses is higher

On the other hand and despite the existence of massive vaccination programmes, HBV continues to be a global public health issue [5-7] that accounts for two billion people that are serologically positive [8] and 325 million individuals with chronic infection [5]. It is the cause of chronic hepatitis, cirrhosis and hepatocellular carcinoma, the latter of which cause approximately 650 thousand deaths per year [6]. Additionally, around 8–16 million cases are estimated to be caused by blood transfusions [9], decrease in treatments and vaccinations [5,8], viral mutations caused by selective pressure of v~~accinations and~~ treatments [6] and access barriers to control programmes [5], which further exacerbates this problem.

in Africa? not in other geographical regions

decrease in treatments?

Escape mutants and

The vaccine is available since 1992, and some of the population borne before that date in not vaccinated

some HBV and HCV

There are ~~relatively few~~ studies on the prevalence of ~~these~~ infections in Colombia, and there is a lack of research on key groups, such as men who have sex with other men (MSM), homeless individuals, lesbians, transgender people, sex workers, injecting drug users and/or inmates. This was evident after applying the following syntax in Scielo *(ab: (hepatitis c)) AND (ab: (hepatitis b)) AND (colombia)* and the following syntax in PubMed *((hepatitis B [Title/Abstract]) AND hepatitis C [Title/Abstract]) AND Colombia*. Despite this fact, several

the definition of the populations including in this study is necessary

previous studies in the field are worth mentioning. An investigation with 619 subjects from

The authors just cited 4 studies carried out in Colombia

four departments in Colombia found the following prevalence levels: HBsAg 5.66%, being

Or the idea was just cited the studies in key groups ?

statistically higher in Magdalena (8.39%) and without association to age or sex; anti-HBc was 28.43% with statistical differences in line with origin, sex and age, which were higher in men (34.36%), people older than 50 years (51.85%) and individuals from the Amazon

w/o any inclusion criteria?

(31.61%) [10]. A screening conducted on 3,369 subjects for HBV and 1,450 for HCV in a public health service provider (IPS by its acronym in Spanish) in Medellín, found prevalence levels of 1.7% for the first round and 0.6% for the second round. Both were associated with age (higher in ages above 40 years) and HBV was found to present a higher incidence rate in men [11]. In 65,535 donors from the same city, the prevalence levels found for HCV and HBV were 0.0% and 0.1% respectively, the latter being statistically higher in men [12]. From the 39,825 donors studied from Montería, 0.32% had HBV and 0.04% had HCV, highlighting statistical association with age and sex, but unlike other studies, the highest proportion, in this case, was found in women [13].

What about the studies of HCV infection performed in PID in different cities in Colombia??

The results of the studies on the general and healthy population without associated risks, are

General population ? none of this study were carried out in general population

high and heterogeneous. This suggests that, in risk groups, this data might be even higher. Learning about the prevalence of both infections in different groups of the community is a

public health priority in Colombia, as the country does not have active epidemiological surveillance programmes in place to combat these infections. Interestingly, these are treatable chronic infections, and their transmission can be controlled given the high efficacy of available antivirals [14].

The objective of this study was to compare the prevalence of HBV, HCV and associated factors in key groups who attended a health care provider (HCP) in Colombia in 2019.

Materials and Methods

Type of study

Multiple-group ecological research study

Study population

A total of 5,771 individuals who attended the Fundación Antioqueña de Infectología (FAI) were included in the study in the period July to December 2019. The groups comprised individuals from the general population (2,624), MSM (1,100), homeless individuals (1,061), lesbians (15), transgender individuals (41), bisexuals (47), inmates (41), sex workers (380), vulnerable young people (260), and injecting drug users (202), who fulfilled the inclusion criteria of being members of one of the key groups of Colombian HIV control policies (with the exception of the general population group that served as a control), participating in intramural or extramural activities of the FAI for STI screening, and being 10 years of age or older (only were included adolescents and adults; after applying these criteria were excluded the individuals who did not sign the assent (for those under 18 years of age) or consent (from

18 years of age) informed, who demanded some type of remuneration to participate in the study, and those whose biological samples were not suitable for carrying out laboratory tests.

Table 1. Distribution of the relevant demographic characteristics of the study population.

N 5.771		%	CI 95%
**Origin**	Medellín	45.75	44.45 – 47.04
	Cali	20.93	18.87 – 21.99
	Bogotá	20.69	19.64 – 21.74
	Quibdó	1.65	1.31-1.98
	Pereira	1.21	0.92-1.50
	Other	9.77	8.99-10.55
**Sex**	Female	41.93	40.65-43.22
	Male	58.07	56.78-59.35
**Age group**	Adolescents (<21 years old)	18.49	17.48-19.50
	21–40 years old	52.82	51.52-54.11
	41–60 years old	20.22	19.18-21.27
	Over 60 years old	8.47	7.75-9.20
**Marital status**	Married/Common-Law Marriage	47.95	46.65-49.24

	Single	51.71	50.41-53.00
	Widow/Separated	0.35	0.19-0.51
**Education level**	None	57.88	56.59-59.16
	Primary	4.49	3.95-5.03
	Secondary	27.50	26.34-28.66
	Superior	10.14	9.35-10.92

The concept of sample or sampling did not apply to the study because the entire population (institutional) was involved. *Stricto sensu* the results are representative of institutionalized populations (in Colombian HCP); however, the results can be extrapolated to groups that share the sociodemographic and sexual risk characteristics described for this population.

The participants were recruited by the FAI in intramural and extramural care campaigns in prisons, areas with high concentration of sex workers, homosocialisation areas and care centres for the homeless were included, from the main cities of Colombia, that is to say Bogotá (capital of Colombia and of the department of Cundinamarca), Medellín (capital of the department of Antioquia), Cali (capital of the department of Valle del Cauca), Quibdó (capital of the department of Chocó) y Pereira (capital of the department of Risaralda).

For the recruitment of the participants, the FAI carries out field work in the following phases: i) The FAI medical team presents the project and the advantages of performing STI screening to leaders of key groups, ii) The leaders of each group socialize the project, the proposal of the fieldwork and propose the sites for the application of the questionnaires and the sampling,

iii) The FAI medical team performs the pre-test advice, explains and obtains the informed consent, provides the doctor's information and the place where the results will be delivered, iv) In positive cases, it is ensured that the participant gets care from their health service and treatment of the infection.

Data collection

The study used primary sources of information based on a survey with sociodemographic and health data (Supplementary material 1) and application of diagnostic tests for both viruses. The survey items were selected based on the experience of the FAI and a review of the literature. The initial version of the instrument was subjected to an appearance validity process to ensure its applicability (according to the criteria of the medical and epidemiological team) and its acceptability (according to the criteria of the subjects of the study groups), with two physicians, two epidemiologists, an infectologist and five people from each study group. Because in this process of the face evaluation no generated changes in the instrument, the validity and relevance of its content to apply to the study population was confirmed.

HCV was detected with SD BIOLONE HCV, immunochromatographic rapid assay that detects antibodies in serum, plasma, or whole blood, by means of a strip coated with recombinant capture antigen with proteins ~~from the HCV genome~~ the HCV proteins Core, NS3, NS4 and NS5. This test has a sensitivity of 100% and a specificity of 99.4%. The OnSite HBsAg Combo Rapid Test (Serum/Plasma/Whole Blood), a lateral flow immunoassay with 100% sensitivity and specificity, was used for HBV.

Selection and information biases were controlled through the application of the eligibility criteria by the medical team, standardisation of extramural work, information and motivation participation campaigns, pre and post-test counselling and the application of diagnostic tests whose risk of false results tended towards zero.

Statistical analysis

Sociodemographic and health variables and risk factors in each group were described with relative frequencies (proportions). The prevalence of HCV and HCV in each study group was determined with a 95% confidence interval. The prevalence of both viruses was compared with the sociodemographic and health variables and risk factors, with the Pearson's Chi square test. Variables that could have been confounding were identified, that is, those presenting an association with the prevalence of HBV or HCV and with another sociodemographic or health variable. A multivariate logistical regression model was performed with these variables for the HBV and another for the HCV, with the dual purpose of excluding confounders and identifying the explanatory variables of the prevalence of both in the study population. Dummy variables were constructed in the logistical regression models, defining the lowest prevalence as the reference group, and a Hosmer–Lemeshow goodness-of-fit test was used to evaluate model fit. Analyses were performed using SPSS 25.0 software with 95% confidence.

Ethical aspects

The study was approved by the FAI Scientific Committee, who applied the guidelines of resolution 8430 of the Colombian Ministry of Health for research with vulnerable groups. The study was considered to have greater-than-minimal risk and the recommendations of the

World Health Organisation (WHO) were to obtain informed consent and guarantee confidentiality, advice, quality of results and referral to health services for treatment- which were thoroughly followed.

Results

Table 2 describes the sociodemographic and health characteristics of the study groups. There was a higher proportion of individuals from Medellín and Cali and a higher proportion of individuals who were under 40 years of age who had little or no schooling (except for vulnerable youth and inmates). There was also high heterogeneity regarding sex distribution, marital status, health affiliation and other health variables in the study groups.

Table 2. Percentage Distribution (%) of the Sociodemographic and Health Features of the Study Groups.

Feature	General	MSM	Homeles s person	Sex worker	Vulnerabl e youth	Injecting drug user	Bisexual	Transsexu al	Lesbian	Inmate
**N**	**2.624**	**1.100**	**1.061**	**380**	**260**	**202**	**47**	**41**	**15**	**41**
**Origin**										
Medellín	41.8	90.5	19.4	11.6	83.1	18.3	63.8	24.4	26.7	0.0
Cali	6.0	3.2	59.8	68.2	0.4	54.5	0.0	24.4	6.7	0.0
Bogotá	30.4	4.6	20.4	20.0	2.7	0.0	29.8	51.2	66.7	0.0
Quibdó	2.9	0.1	0.0	0.0	6.9	0.0	0.0	0.0	0.0	0.0
Pereira	18.8	1.6	0.4	0.3	6.9	27.2	6.4	0.0	0.0	100.0
**Sex**										
Female	58.5	12.6	14.1	94.7	57.3	17.3	38.3	36.6	93.3	12.2
Male	41.5	87.4	85.9	5.3	42.7	82.7	61.7	63.4	6.7	87.8
**Age group**										
Adolescents (< 21 years)	19.6	24.6	8.5	6.3	46.9	11.9	31.9	2.4	26.7	2.4
21–30 years	30.8	45.0	23.9	35.3	51.9	55.0	53.2	53.7	46.7	43.9
31–40 years	16.0	16.1	23.7	29.5	0.0	26.7	12.8	24.4	20.0	19.5
41–50 years	10.2	6.2	16.7	18.2	0.4	6.4	0.0	2.4	6.7	12.2

51–60 years	9.1	6.4	19.7	8.	0.8	0.0	2.1	9.8	0.0	12.2
Older than 60 years	14.3	1.7	7.5	2.1	0.0	0.0	0.0	7.3	0.0	9.8
**Schooling**										
None	50.9	72.1	63.3	87.1	0.0	73.8	31.9	80.5	73.3	0.0
Incomplete primary	3.0	0.1	0.2	0.0	2.7	18.8	0.0	0.0	0.0	7.3
Complete primary	3.2	1.1	1.1	0.0	6.9	0.0	8.5	0.0	0.0	0.0
Incomplete secondary	0.9	0.3	10.6	1.8	1.5	2.0	2.1	0.0	0.0	9.8
Completed secondary	29.0	20.1	24.8	10.8	26.9	5.4	38.3	17.1	13.3	82.9
Technical	5.0	3.2	0.0	0.3	56.9	0.0	6.4	2.4	13.3	0.0
University	8.0	3.2	0.0	0.0	5.0	0.0	12.8	0.0	0.0	0.0
**Marital status**										
Married—Common Law Marriage	41.1	69.9	49.4	67.4	1.5	43.1	19.1	70.7	46.7	9.8
Single	58.4	30.0	50.4	32.4	98.5	56.9	80.9	26.8	53.3	87.8
Widowed—Separated	0.5	0.1	0.2	0.3	0.0	0.0	0.0	2.4	0.0	2.4
**Ethnicity (self-perceived)**										
Afro-descendent	0.4	0.3	1.8	0.3	0.4	0.0	2.1	0.0	0.0	0.0
White	50.0	71.6	63.1	83.7	0.0	69.8	31.9	80.5%	73.3	0.0
Mestizo	49.6	28.1	35.2	16.1	99.6	30.2	66.0	19.5	26.7	100.0

**Health affiliation**										
Without affiliation	6.0	11.9	20.9	15.8	4.6	12.9	17.0	14.6	13.3	14.6
Subsidised regime	34.3	26.8	52.2	53.2	32.7	53.5	8.5	63.4	13.3	17.1
Contributory regime	59.7	61.3	26.9	31.1	62.7	33.7	74.5	22.0	73.3	68.3
**Other health features**										
Hospitalisation in the last 12 months	12.2	12.0	5.5	6.6	19.0	20.3	10.6	7.5	0.0	2.4
Medication in the last month	23.2	15.3	11.5	13.2	19.4	34.2	21.3	17.5	20.0	2.4
Having received a transfusion or transplant	7.7	5.9	2.5	2.6	11.9	5.9	8.5	5.0	0.0	2.4
Having been vaccinated within the last year	20.1	10.0	7.4	14.5	19.8	8.9	14.9	20.0	6.7	2.4

The table includes the percentages within each group (column percentages).

In all of the study groups, more than 90% of subjects reported risky sexual intercourse. Risks with the lowest prevalence included biological accidents, sexual intercourse with individuals with sexually transmitted infections (STIs) and having an imprisoned sexual partner. The use of hallucinogens was higher amongst homeless individuals, having a new sexual partner was more frequent amongst MSM, vulnerable and bisexual youth and body piercings were more common in lesbians and bisexuals (Table 3).

Table 3. Percentage Distribution (%) of Sexual Risk Factors amongst the Study Groups.

Factor	General	MSM	Homeless person	Sex worker	Vulnerable youth	Injecting drug user	Bisexual	Transsexual	Lesbian	Inmate
**Use of hallucinogens**	24.6	35.2	81.7	40.5	23.0	93.6	40.4	42.5	33.3	36.6
**Risky sexual behaviour**	96.7	96.8	99.9	100.0	90.8	100.0	91.5	97.6	100.0	100.0
**Intercourse with key group**	13.8	44.9	27.3	15.8	13.9	34.2	55.3	57.5	53.3	22.0
**Intercourse with people with STIs**	8.1	8.1	3.1	1.6	11.9	3.0	8.5	7.5	0.0	0.0
**New sexual partner within the last six (6) months**	35.4	52.5	26.2	40.3	55.6	30.7	59.6	40.0	33.3	56.1
**Having received psychoactive substances or money in exchange for sexual intercourse**	10.0	17.1	14.1	54.7	14.7	18.8	17.0	47.5	0.0	7.3
**In the last twelve (12) months the subject or his sexual partner has been deprived of liberty**	12.4	6.7	11.3	5.3	11.5	8.9	8.5	5.0	0.0	Non-applicable
**In the last twelve (12) months the subject or his sexual partner had piercings made**	23.2	22.3	8.6	11.1	25.4	13.4	31.9	10.0	46.7	19.5

**In the last twelve (12) months the subject has suffered biohazard accidents**	7.1	4.9	2.0	0.3	11.9	2.5	8.5	0.0	0.0	0.0

The table includes the percentages within each group (column percentages).

Neither the prevalence of HBV, nor that of HCV presented statistical associations with marital status, receiving a blood transfusion or organ transplantation, receiving vaccination in the last year, identifying risky sexual relationships, having sexual relations with individuals diagnosed with STIs, changing sexual partners during the last six months, receiving money or psychoactive substances in exchange for money, being deprived of liberty (the study subject or his sexual partner), having body piercings, or biohazard accidents in the last year (Chi square $p > 0.05$).

The prevalence of HBV was statistically higher amongst injecting drug users (5.94%), in men (0.63%), in Afro-descendants (2.78%) and in people without health affiliation (1.11%). On the other hand, the prevalence of HCV was statistically higher amongst injecting drug users (45.54%), men (3.91%), people aged 21 to 30 years (3.85%) and 31 to 40 years (4.04%), uneducated individuals (3.68%), Caucasians (3.56%), those without health affiliations (4.29%), or those from the subsidised regime (4.31%), people who use hallucinogens during sexual intercourse (5.71%) and amongst those who have sex with people from key groups (4.56%) (Table 4).

Table 4. Specific Prevalence of HBV and HCV According to Study Group and Sociodemographic and Health Characteristics.

Variable	Levels	N	HBV		HCV	
			% (n)	CI 95%	% (n)	CI 95%
Group	General	2.624	0.15 (4)	0.04–0.39	0.27 (7)	0.05–0.48
	MSM	1.100	0.27 (3)	0.06–0.79	2.09 (23)	1.20–2.98 [a*]
	Homeless	1.061	0.37 (4)	0.10–0.96	2.17 (23)	1.24–3.09 [a*]
	Sex workers	380	0.26 (1)	0.01–1.46	No cases	—
	Vulnerable youth	260	0.39 (1)	0.01–2.12	No cases	—
	Injecting drug users	202	5.94(12)	2.43–9.45[b**]	45.54 (92)	38.43–52.66[b**]
Origin	Medellín	2.640	0.15 (4)	0.04–0.39	0.42 (11)	0.15–0.68
	Cali	1.208	0.25 (3)	0.05–.072	5.46 (66)	4.14–6.79[b**]

	Bogotá	1.194	0.34 (4)	0.09–0.86	0.50 (6)	0.06–0.95
	Others	634	2.21 (14)	0.98–3.43 b**	9.78 (62)	7.39–12.17 b**
**Sex**	Female	2.420	0.16 (4)	0.05–0.42	0.58 (14)	0.26–0.90
	Male	3.351	0.63 (21)	0.35–0.91 a*	3.91 (131)	3.24–4.58 b**
**Age group**	Adolescents (< 21 years)	1.067	0.09 (1)	0.0–0.52	0.94 (10)	0.31–1.56
	21–30 years	2.008	0.50 (10)	0.17–0.83	3.85 (77)	2.97–4.70b**
	31–40 years	1.040	0.63 (7)	0.13–1.22	4.04 (42)	2.79–5.28 b**
	41–50 years	603	0.33 (2)	0.04–1.19	1.99 (12)	0.79–3.19 a*
	51–60 years	564	0.71 (4)	0.19–1.81	0.18 (1)	0.00–0.98
	Older than 60 years	489	0.20 (1)	0.00–1.13	0.61 (3)	0.13–1.78
**Schooling**	None	3.340	0.63 (21)	0.35–0.91	3.68 (123)	3.03–4.34 b**
	Incomplete primary	130	No cases	—	5.38 (7)	1.52–9.65 b**
	Complete primary	129	0.77 (1)	0.02–4.24	No cases	—
	Incomplete secondary	158	No cases	—	1.27 (2)	0.15–1.50
	Completed secondary	1429	0.21 (3)	0.04–0.61	0.77 (11)	0.28–1.26
	University	263	No cases	—	0.76 (2)	0.09–2.72
**Ethnicity**	Afro-descendent	36	2.78 (1)	0.07–14.53b**	No cases	—
	White	3.286	0.55 (18)	0.28–0.82	3.56 (117)	2.91–4.21**
	Mestizo	2.449	0.25 (6)	0.03–0.46	1.14 (28)	0.70–1.59
**Health affiliation**	Without affiliation	630	1.11 (7)	0.21–2.01 a**	4.29 (27)	2.63–5.95 b**
	Subsidised regime	2.183	0.64 (14)	0.28–1.00	4.31 (94)	3.43–5.18 b**
	Contributory regime	2.958	0.13 (4)	0.04–0.35	0.81 (24)	0.47–1.15
**Others**	Hospitalisation	627	0.48 (3)	0.10–1.39	4.78 (30)	3.03–6.54 b**
	Medication consumption	1.081	0.46 (5)	0.15–1.08	4.90 (53)	3.57–6.24 b**

	Use of hallucinogens during sex	2.342	0.77 (18)	0.39–1.14	5.72 (134)	4.76–6.68 [b**]
	Identified risk relationship	5.619	0.43 (24)	0.25–0.61	2.58 (145)	2.16–3.00 [a*]
	Sex with key groups	1.360	0.74 (10)	0.24–1.23	4.56 (62)	3.41–5.70 [b**]

No cases of HBV or HCV were found in the following groups: bisexual, transgender, lesbian, inmates, residents of Quibdó, technical training.

[a] Statistically higher prevalence than the lowest prevalence subgroup. [b] Statistically higher prevalence than all subgroups.[*] $p < 0.05$ [**] $p < 0.01$.

Only three explanatory variables were identified in the multivariate HBV model: study group, city of origin and type of health affiliation. The probability for HBV in MSM, homeless individuals and vulnerable youth was five times higher than that found in the general population; amongst sex workers it was 3.7 and it was 33.7 amongst injecting drug users. It was 3.5 amongst individuals without health affiliations compared to those from people affiliated with the contributory scheme. For HCV, the explanatory variables were group, origin, sex, age group, health affiliation and use of drugs and hallucinogens during sexual intercourse, with higher odds ratios in MSM (25.6), injecting drug users (77.5), inhabitants of Cali (17.4), men (2.1), people between 31 and 40 years of age (2.9), those affiliated with the subsidised regime (5.9) and those who use hallucinogens during sexual intercourse (Table 5).

Table 5. Multivariate Logistic Regression Models to Identify Potentially Explanatory Factors for the Prevalence of HBV and HCV

	Wald	p	Odds ratio (CI 95%)
**HBV model variables**			
**Group**	**31.55**	**0.000**	
MSM/general population	3.73	0.048	5.3 (1.00–28.30)

Homeless/general population	4.09	0.043	5.1 (1.05–25.13)
Sex workers/general population	1.18	0.277	3.7 (0.35–38.48)
Vulnerable youth/general population	2.18	0.140	5.5 (0.57–52.32)
Injecting drug users/general population	30.36	0.000	33.7 (9.65–117.86)
**Origin**	**20.24**	**0.000**	
Cali/Medellín	0.05	0.823	0.8 (0.15–4.49)
Bogotá/Medellín	2.16	0.141	3.3 (0.67–16.54)
Others/Medellín	12.77	0.000	12.4 (3.11–49.10)
**Health affiliation**	**3.56**	**0.047**	
Unaffiliated/Contributory	3.35	0.047	3.5 (1.00–13.39)
Subsidised/Contributory	2.54	0.111	2.6 (0.81–8.20)
**HCV model variables**			
**Group**	**144.18**	**0.000**	
MSM/general population	36.24	0.000	25.6 (8.95–73.70)
Homeless/general population	4.46	0.035	3.1 (1.08–9.10)
Injecting drug users/general population	71.70	0.000	77.5 (28.3–212.2)
**Origin**	**108.03**	**0.000**	
Cali/Medellín	46.86	0.000	17.4 (7.70–39.3)
Bogotá/Medellín	12.93	0.000	7.5 (2.43–22.21)
Others/Medellín	104.28	0.000	98.3 (40.7–237.0)
**Sex** (Male/Female)	**3.98**	**0.046**	2.1 (1.01–4.34)
**Age group**	**9.83**	**0.048**	
<21 / >60 years	0.11	0.736	1.3 (0.29–5.74)
21–30 / >60 years	2.43	0.049	2.8 (1.00–10.3)
31–40 / >60 years	2.52	0.048	2.9 (1.00–10.86)
41–50 / >60 years	0.77	0.380	1.9 (0.46–7.69)
51–60 / >60 years	0.83	0.364	0.3 (0.03–3.45)
**Health affiliation**	**34.41**	**0.000**	

Unaffiliated/Contributory	6.79	0.009	2.9 (1.30–6.35)
Subsidised/Contributory	33.99	0.000	5.9 (3.26–10.77)
**Medications** (Yes/No)	**10.38**	**0.001**	2.4 (1.40–3.96)
**Use of hallucinogens during sex** (Yes/No)	**16.01**	**0.000**	5.3 (2.33–11.90)

Discussion

Considering that there was a large population with subjects from different cities within the country,

high diagnostic validity ? the authors could demonstrated of the sensitivity of these rapid tests?

applying a test with high diagnostic validity resulted in the prevalence of HBV and HCV to be 0.15%

Unfortunately the authors could not analyzed the samples using molecular techniques in order to confirm the results

and 0.27% amongst the general population, respectively. Said prevalence was 0.27% and 2.09% amongst MSM; 0.37% and 2.17% amongst homeless; 0.26% and 0.0% amongst sex workers; 0.39% and 0.0% amongst vulnerable youth; and 5.94% and 45.54% amongst injecting drug users. The explanatory variables for both infections study group were the origin and type of health affiliation. This is of great clinical and public health significance as it reveals the large number of people who are at risk for cirrhosis, carcinoma, and even death in the event of not receiving adequate treatment and follow-up for their condition, while also demonstrating a high risk of transmission of both viruses within the country.

HBV and HCV prevalence in the general population was 0.15% and 0.27%, which is lower than that

The data obtained using rapid test are not comparable to Elisa test

reported worldwide (approximately 1%) and in Latin America (approximately 0.7%). This highlights an important improvement for this group, although these low frequencies, in the context of issues accessing treatment in Colombia, should draw attention to the presence of asymptomatic carriers who are at high risk of contracting cirrhosis or liver cancer [1,2].

Prevalence of HBV was 0.27% and that of HCV was 2.09% amongst MSM. Studies in other countries show varying results for HCV ranging from 1% in Peru [15] to 22.7% in Australia [16]. Although hepatitis B has long been considered a sexually transmitted infection, the role of sexual contact in the transmission of hepatitis C has become less clear [17]. In this sense, several studies have concluded that heterosexual transmission of HCV is inefficient, whereas other molecular epidemiological studies have

identified HCV transmission clusters in MSM networks [18]. The causes behind increased sexual transmission of HCV in MSM are complex and result from the interaction of several factors, including unprotected anal intercourse, which represents a high risk of infection for the receptive partner. The practice of serosorting (participating in sexual activities with individuals with the same HIV status) has become increasingly common amongst MSM and constitutes an unsafe practice as it does not prevent other STIs, including HCV. Sexual practices such as *fisting* and sharing of sex toys can cause extreme dilation of anal tissue and micro-bleeding during sexual activity. Group sex practices can also cause injury to the mucosal surfaces and rectal bleeding. Recreational drug use during sexual intercourse generates disinhibition and increased sexual excitement and lowers the perception of risk, which adds to the likelihood of contracting HIV and/or other STIs that may increase biological vulnerability [17,18]. In light of these facts, it is essential to promote safe sexual practices in this population as they generate significant impacts on infection not only by HBV and HCV, but also on HIV and other STIs.

Prevalence of HBV was 0.26% and 0.0% for HCV amongst sex workers. In a study carried out on sex workers in Brazil, the prevalence found for HBV was 17.1% and that for HCV was 0.7% [19]. In a meta-analysis of the prevalence of HCV in key populations in Latin America and the Caribbean, it was found that the frequency amongst sex workers was 2% [20]. Conversely, a study on sex workers in Vietnam found that the prevalence of HCV ranges between 8.8% and 30.4%; however, injecting drug use was the main cause of infection [21]. The few cases of HCV amongst sex workers and the high prevalence amongst injecting drug users, reaffirm the hypothesis of the low HCV transmission efficiency in heterosexual or homosexual female intercourse [22]. Further research must be carried out to explain the low prevalence of HBV and the difference with other countries in the region, such as Brazil with 17.1% [19], Argentina with 14.4% [23], Bolivia with 13.1% [24] and Venezuela with 13.8% [25].

The study design and the technique for detection of infection marker are similar ??

Prevalence of HBV was 0.37% and that of HCV was 2.17% amongst homeless people. According to DANE (National Administrative Department of Statistics by its Spanish acronym) figures, there are

approximately 22,790 homeless individuals in the main cities of Colombia. Studies that quantify both infections in this population are scarce; however, a study was found in California, United States which reported the prevalence of HBV as 1.17% and of 41.7% for HCV [26]. Research on HCV in this population was more prolific. In this sense, a 2012 meta-analysis reported that HCV prevalence ranges from 3.9% to 36.2% and combined prevalence of infection was 20.3% [27]. Another meta-analysis published in 2018 found a combined prevalence of 28% [28]. Discrepancies in results can be attributed to differences in sampling methods, diagnostic tests used (some studies use self-reporting techniques), criteria used to define the status of the homeless and inclusion of the determinants of infection. Beyond these differences between studies, it is recommended that marginalised groups undergo concurrent tests which, in addition to tracking HBV and HCV, can detect tuberculosis and HIV. In cases of infection, patients should be helped to overcome barriers to completing diagnostic exams and treatments and helped to plan for transportation, housing, nutrition and, where required, migration to their cities of origin.

The prevalence found for HBV was 0.39% and that of HCV was 0.0% amongst vulnerable youth. Vulnerable youth in Colombia include a population group that is more exposed to abuse of their fundamental rights, exclusion, poverty, inequality and various sorts of violence. Data on HBV prevalence amongst adolescents or young individuals are scarce and the figures on annual mortality are not yet known [29]. Compared to adults, there has been little focus on diagnosis and treatment within this population, partly because most patients are in the immune-tolerant phase and do not require treatment [29]. In this sense, it is necessary to carry out seroepidemiological studies stratified by age to assess the prevalence of HBsAg in different populations with estimates on the burden, morbidity, mortality, and need for treatment by region. Considering that vertical transmission (mother-to-child) and horizontal transmission during early childhood are the main routes of transmission of HBV and that they are responsible for most chronic infections, it is important to seek universal immunisation at birth and during

childhood, mainly in vulnerable populations as an effective strategy for lowering the incidence of new infections [29]. There are no data of vaccine programs in this population?

The prevalence of HBV amongst injecting drug users was 5.94% and that of HCV was 45.54%. Research on this topic is scarce in Latin American countries; however, a research study conducted in Brazil revealed that the prevalence of HCV in this population group is 35.5% [30]. A systematic review on the prevalence of these types of hepatitis amongst injecting drug users in European countries determined that national estimates of HBV ranged from 0.5% in Croatia, Hungary and Ireland to 6.3% in Portugal. For HCV, findings ranged from 13.8% in Malta to 84.3% in Portugal. Prevalence within this group was also reported to be higher than that found in the general population, men who have sex with men, inmates and migrants [31]. Another systematic review in Iran reported that the prevalence of HCV amongst injecting drug users was 47% [32]. The lower proportion of HBV infection can be explained by the availability of

vaccination of the study population was included in the questionary?

the vaccine and because the risk of chronification of infection is relatively low when it is acquired during adolescence or adulthood [33]. The consistency of the findings between studies from different countries confirm that the population of injecting drug users may well be the group with the highest risk for HBV and HCV infection; therefore, they are proposed to be sentinel groups for monitoring infection and characterising circulating genotypes. Regarding measures to reduce infection, the establishment of centres for the distribution of disposable syringes, the performance of free diagnostic tests, the distribution of condoms and detoxification treatments such as the one carried out with methadone, are suggested.

The study group, the origin and the type of health affiliation were the explanatory variables for both infections. This information is key because all WHO member states have committed to a global reduction of hepatitis-related deaths by 65% and new infections by 90%, by 2030 [34]. In order to achieve this goal, it is necessary to focus on actions within the country by region and by special groups, such as injecting drug users or MSM and to eliminate barriers to universal and timely access to health services.

Regarding the type of intervention, massive testing, universal vaccination for hepatitis B, improvement of access to antiviral treatments and reduction of their cost have been proposed. However, nationwide research studies are necessary to ensure evidence-based decision-making processes are taking place regarding the type of interventions that will provide the greatest public health benefits. In China, for example, investing in comprehensive HBV programming is expected to generate savings of more than $ 1.5 for every $ 1 spent by 2030 (34).

one of the limitations of this study is the lack of informations of HBV and HCV prevalence in the country and is some risk populations

The limitations of this research include the temporal bias of the exposed statistical relationships, the absence of causal associations and the non-discrimination between chronic and active infections. Despite these limitations, the importance of prevalence studies should be highlighted as a key element for the initiation and/or orientation of all types of clinical, epidemiological, public health and research actions.

Conclusion

High prevalence??? just the HCV prevalence in PID

A ~~high~~ prevalence of both viral infections was evident in the population, with much higher rates within the key groups. The main associated factors identified related to origin and type of health affiliation demonstrated a double vulnerability, that is, belonging to groups that are discriminated and excluded from various health policies and living under unfavourable socioeconomic conditions that prevent proper affiliation and health care. These results corroborate the need for improving active epidemiological surveillance; increasing resources for prevention, diagnosis and treatment; addressing structural problems of health service delivery; promoting the inclusion of key groups in the management health policies; and improving the quantity and quality of scientific evidence in this field.

Acknowledgements

Walter Osorio de la Fundación Antioqueña de Infectología.

References

1. Organización Mundial de la Salud (OMS). Los datos más recientes ponen de relieve la necesidad de actuar urgentemente a nivel mundial contra las hepatitis. 2019. Available from: https://www.who.int/es/news-room/detail/21-04-201-new-hepatitis-data-highlight-need-forurgent-global-response. Accessed April 2020.
2. World Health Organization. Global Hepatitis Report 2017. 2017. Available from: https://apps.who.int/iris/bitstream/handle/10665/255016/9789241565455-eng. Accessed April 2020.
3. Rustgi VK. The epidemiology of hepatitis C infection in the United States. J Gastroenterol. 2007;42(7): 513-521. doi:10.1007/s00535-007-2064-6.
4. Westbrook RH, Dusheiko G. Natural history of hepatitis C. J Hepatol. 2014;61(Suppl 1): S58-S68. doi: 10.1016/j.jhep.2014.07.012.
5. Organización Mundial de la Salud (OMS). Los datos más recientes ponen de relieve la necesidad de actuar urgentemente a nivel mundial contra las hepatitis. 2017. Available from: https://www.who.int/es/news-room/detail/21-04-2017-new-hepatitis-data-highlight-need-for-urgent-global-response. Accessed April 2020.
6. Farooq A, Waheed U, Zaheer HA, Aldakheel F, Alduraywish S, Arshad M. Detection of HBsAg mutants in the blood donor population of Pakistan. PLoS ONE. 2017;12(11): 1-11. doi: 10.1371/journal.pone.0188066
7. El-Ghitany EM, Farghaly AG. Evaluation of commercialized rapid diagnostic testing for some Hepatitis B biomarkers in an area of intermediate endemicity. J Virol Methods. 2013;194(1-2): 190-193. doi: 10.1016/j.jviromet.2013.08.026.
8. Randrianirina F, Carod J-F, Ratsima E, Chrétien J-B, Richard V, Talarmin A. Evaluation of the performance of four rapid tests for detection of hepatitis B surface antigen in Antananarivo, Madagascar. J Virol Methods. 2008;151(2): 294-297. doi: 10.1016/j.jviromet.2008.03.019.

9. Dogbe EE, Arthur F. Diagnostic accuracy of blood centers in the screening of blood donors for viral markers. Pan Afr Med J. 2015;20: 119. doi: 10.11604/pamj.2015.20.119.5263
10. Alvarado-Mora MV, Fernandez MF, Gomes-Gouvêa MS, de Azevedo Neto RS, Carrilho FJ, Pinho JR. Hepatitis B (HBV), hepatitis C (HCV) and hepatitis delta (HDV) viruses in the Colombian population--how is the epidemiological situation? PLoS One. 2011;6(4): e18888. doi: 10.1371/journal.pone.0018888.
11. Callejas-Rojo F, Serna-Quintana G, Cardona-Arias J. Frecuencia de reactividad a la prueba VDRL y de positividad para Chlamydia spp, VIH y virus de la hepatitis B y C en una institución prestadora de servicios de la salud de Medellín, 2015. CES Med. 2017;31(1): 27-37.
12. Bedoya J, Cortés M, Cardona-Arias J. Seroprevalencia de marcadores de infecciones transmisibles por vía transfusional en banco de sangre de Colombia. Rev Saúde Pública. 2012;46(6): 950-959.
13. Ruiz Mendoza L, Villegas Gracia R, Cardona-Arias J. Prevalencia de agentes transmisibles por transfusión y factores asociados en un banco de sangre de Córdoba-Colombia 2014-2016. Rev. UDCA. 2018;21(2): 297-308.
14. Abad S, Vega A, Rincón D, Hernández E, Mérida E, Macías N, et al. Eficacia de los antivirales de acción directa en la infección por el virus de la hepatitis C en pacientes en hemodiálisis. Nefrología. 2017;37(2): 158-163. doi: 10.1016/j.nefro.2016.10.003.
15. Hyams KC, Phillips IA, Moran AY, Tejada A, Wignall FS, Escamilla J. Seroprevalence of hepatitis C antibody in Peru. J Med Virol. 1992;37(2): 127-131. doi: 10.1002/jmv.1890370210.
16. Fairley CK, Leslie DE, Nicholson S, Gust ID. Epidemiology and hepatitis C virus in Victoria. Med J Aust. 1990;153(5): 271-273. doi: 10.5694/j.1326-5377.1990.tb136899.x
17. Gorgos L. Sexual transmission of viral hepatitis. Infect Dis Clin N Am. 2013;27(4): 811-836. 10.1016/j.idc.2013.08.002.
18. Bradshaw D, Matthews G, Danta M. Sexually transmitted hepatitis C infection: the new epidemic in MSM? Curr Opin Infect Dis. 2013;26(1): 66-72. doi: 10.1097/QCO.0b013e32835c2120.

19. Matos MA, França DDDS, Carneiro MADS, Martins RMB, Kerr LRFS, Caetano KAA, et al. Viral hepatitis in female sex workers using the Respondent-Driven Sampling. Rev Saude Publica. 2017;51: 65. doi: 10.1590/S1518-8787.2017051006540.

20. Alonso M, Gutzman A, Mazin R, Pinzon CE, Reveiz L, Ghidinelli M. Hepatitis C in key populations in Latin America and the Caribbean: systematic review and meta-analysis. Int J Public Health. 2015;60(7): 789-798. doi: 10.1007/s00038-015-0708-5.

21. Le LN, O'Connor S, Tran TH, Maher L, Kaldor J, Sabin K, et al. High hepatitis C virus infection among female sex workers in Viet Nam: strong correlation with HIV and IV drug use. Western Pac Surveill Response J. 2019;10(3): 9-18. doi: 10.5365/wpsar.2019.10.1.002.

22. Baden R, Rockstroh JK, Buti M. Natural history and management of hepatitis C: Does sex play a role? J Infect Dis. 2014; 209(Suppl 3): S81-S85. doi: 10.1093/infdis/jiu057.

23. Pando MA, Berini C, Bibini M, Fernández M, Reinaga E, Maulen S, et al. Prevalence of HIV and other sexually transmitted infections among female commercial sex workers in Argentina. Am J Trop Med Hyg. 2006;74(2): 233-238.

24. León P, Venegas E, Bengoechea L, Rojas E, López JA, Elola C, Echevarría JM. Prevalence of infections by hepatitis B, C, D and E viruses in Bolivia. Rev Panam Salud Publica. 1999;5(3): 144-151. doi: 10.1590/s1020-49891999000300002.

25. Camejo MI, Mata G, Díaz M. Prevalence of hepatitis B, hepatitis C and syphilis in female sex workers in Venezuela. Rev Saude Publica. 2003;37(3): 339-244. doi: 10.1590/s0034-89102003000300012.

26. Cheung RC, Hanson AK, Maganti K, Keeffe EB, Matsui SM. Viral hepatitis and other infectious diseases in a homeless population. J Clin Gastroenterol. 2002;34(4): 476-480. doi: 10.1097/00004836-200204000-00021

27. Beijer U, Wolf A, Fazel S. Prevalence of tuberculosis, hepatitis C virus, and HIV in homeless people: a systematic review and meta-analysis. Lancet Infect Dis. 2012;12(11): 859-870. doi: 10.1016/S1473-3099(12)70177-9

28. Hakobyan S, Sepehry AA, Nikoo N, Khachatryan D, Nikoo M, Song MJ, et al. An update of Hepatitis C prevalence rates in homeless adults after Hepatitis C treatment paradigm change: A systematic review and meta-analysis. Med Res Arch. 2018;6(1): 1-15. doi: 10.18103/mra.v6i1.1596.
29. Indolfi G, Easterbrook P, Dusheiko G, Siberry G, Chang MH, Thorne C, et al. Hepatitis B virus infection in children and adolescents. Lancet Gastroenterol Hepatol. 2019;4(6): 466-476. doi: 10.1016/S2468-1253(19)30042-1
30. Silva MB, Andrade TM, Silva LK, Rodart IF, Lopes GB, Carmo TM, et al. Prevalence and genotypes of hepatitis C virus among Injecting drug users from Salvador-BA, Brazil. Mem Inst Oswaldo Cruz. 2010;105(3): 299-303. doi: 10.1590/s0074-02762010000300009.
31. European Centre for Disease Prevention and Control. Systematic review on hepatitis B and C prevalence in the EU/EEA. Stockholm: ECDC; 2016. Available from: https://www.ecdc.europa.eu/sites/portal/files/media/en/publications/Publications/systematic-review-hepatitis-B-C-prevalence.pdf. Accessed April 2020.
32. Behzadifar M, Behzadifar M, Bragazzi NL. A systematic review and meta-analysis of the prevalence of hepatitis C virus infection in people who inject drugs in Iran. BMC Public Health. 2020;20(1): 62. doi: 10.1186/s12889-020-8175-1.
33. Edmunds WJ, Medley GF, Nokes DJ, Hall AJ, Whittle HC. The influence of age on the development of the hepatitis B carrier state. Proc Biol Sci. 1993;253:197-201.
34. Cooke GS, Andrieux-Meyer I, Applegate TL, Atun R, Burry JR, Cheinquer H, et al. Accelerating the elimination of viral hepatitis: a Lancet Gastroenterology & Hepatology Commission. Lancet Gastroenterol Hepatol. 2019;4(2): 135-184. doi: 10.1016/S2468-1253(18)30270-X.
